# Fermentative nitrite ammonifiers are abundant in soils and ecologically distinct from NrfA-dependent ammonifiers

**DOI:** 10.1093/ismeco/ycag144

**Published:** 2026-05-23

**Authors:** Yuyan Teng, Aurélien Saghaï

**Affiliations:** Department of Forest Mycology and Plant Pathology, Swedish University of Agricultural Sciences, 75651 Uppsala, Sweden; Department of Forest Mycology and Plant Pathology, Swedish University of Agricultural Sciences, 75651 Uppsala, Sweden

**Keywords:** nitrogen cycle, *nirB*, metagenomics, phylogenetics

## Abstract

Microorganisms can use different enzymes to perform nitrite ammonification, the reduction of nitrite to ammonium, an important process to retain nitrogen in soils. Yet, the organisms mediating this process and their distribution in terrestrial ecosystems remain poorly resolved. Here, we determined the phylogenetic diversity of bacteria performing fermentative nitrite ammonification via the NAD(P)H-dependent nitrite reductase NirB, assessed their distribution across terrestrial ecosystems, and identified their environmental preferences. We found that these organisms are broadly distributed, spanning 29 phyla including Bacillota, Pseudomonadota and Actinomycetota. Screening 1587 globally distributed soil metagenomes using a phylogeny-based approach revealed that fermentative nitrite ammonifiers are ubiquitous across biomes and particularly abundant in Mediterranean forests and desert soils. In these ecosystems, they outnumbered NrfA-dependent ammonifiers, the best characterized ammonifier group to date, suggesting distinct ecological niches for the two groups. Consistent with this, random forest modelling revealed a negative relationship between fermentative nitrite ammonifiers and the carbon-to-nitrate ratio, which contrasts with a preference for high carbon-to-nitrate conditions in NrfA-dependent ammonifiers. However, moisture and salinity emerged as the strongest predictors of the abundance of fermentative nitrite ammonifiers, indicating a high tolerance to osmotic stress in this group. Overall, our results demonstrate that fermentative nitrite ammonifiers are both phylogenetically diverse and environmentally widespread, calling for future efforts to determine the conditions under which they contribute to nitrogen retention in soils.

Nitrogen (N) often limits primary production in terrestrial ecosystems, with its bioavailability governed by microbial processes [1]. One such process, nitrite ammonification, acts like a short circuit in the N cycle, transforming nitrite back into ammonium without the release of substantial gaseous intermediates. Nitrite ammonification is beneficial in N limited ecosystems such as tundra or in agricultural soils where it is desirable to improve nitrogen use efficiency [2], as ammonium can bind to negatively charged surfaces in the soil or be assimilated by plants or microorganisms.

Despite recent advances on the diversity and ecology of NrfA-dependent ammonifiers [3], ammonifiers remain one of the least studied agents of the N cycle, especially those relying on other ammonifying enzymes such as the NAD(P)H-dependent nitrite reductase. This enzyme, usually designated NirB, is involved in several physiological functions, including nitrite detoxification and assimilatory reduction of nitrite to ammonium [4]. In *E. coli*, NirB can also be used to regenerate the NAD^+^ pools for glycolysis during fermentative growth, resulting in the generation of one extra ATP molecule by substrate level phosphorylation for each molecule of acetate generated [5]. Although the majority of the resultant ammonium is thought to remain in the cell, some is released to the environment [6, 7], thereby providing a possible means for reactive N to be retained in soils. Despite *nirB* being abundant in soil metagenomes [8], fermentative nitrite ammonifiers are mostly studied in the context of enterobacteria and the abundance, diversity and ecology of these organisms in terrestrial ecosystems is not known. To fill this knowledge gap, we first built a reference phylogeny for NirB by screening publicly available prokaryotic and fungal genomes and determined the genetic potential for fermentation in the bacterial genomes. We then assessed the abundance of fermentative nitrite ammonifiers in 1587 globally distributed soil metagenomes and identified their environmental preferences using random forest modelling.

The genome search resulted in 981 non-redundant structurally diverse NirB sequences spanning 37 bacterial phyla (*n* = 839 genomes) and 2 fungal phyla (*n* = 127 genomes). Bacterial genomes carrying more than one *nirB* copy (*n* = 2 or 3; 8% of the genomes) were exclusively found in Pseudomonadota and Bacillota. All sequences contained FAD and NADH binding domains in the N-terminal region, two Cys-X-X-Cys motifs corresponding to the binding site of an iron sulfur cluster, as well as a siroheme binding site (Cys-X-X-X-Cys) at the C-terminal region [9]. Phylogenetic reconstruction showed that bacterial and fungal sequences formed distinct clades (Fig. 1). While some clades were largely restricted to specific phyla such as Bacillota, Pseudomonadota and Actinomycetota, others contained representatives derived from much more phylogenetically diverse organisms.

**Figure 1:**
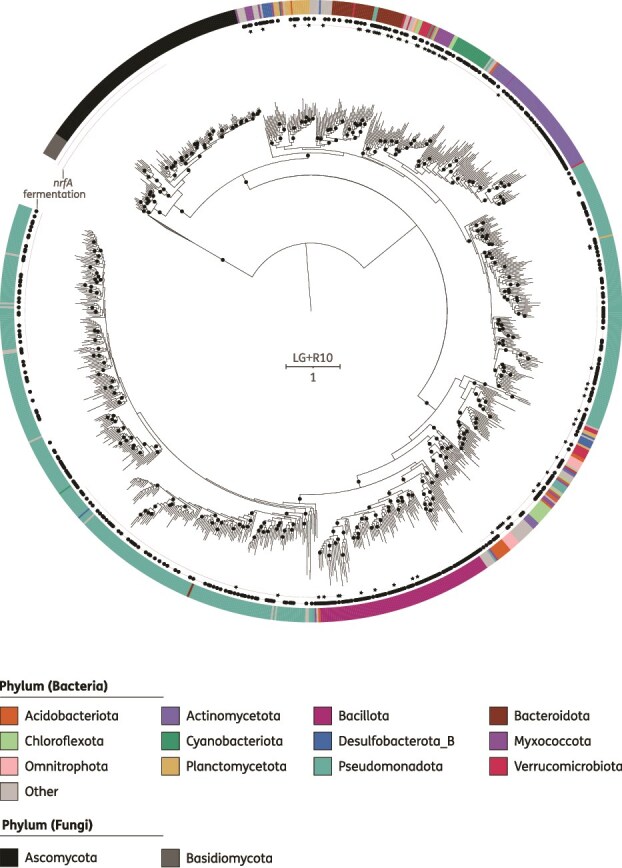
**Maximum likelihood phylogeny of 981 bacterial and fungal NirB sequences inferred from the alignment of 857 amino acid positions.** The presence of *nrfA* in the genomes corresponding to each NirB sequence is indicated by stars in the inner ring. Sequences obtained from genomes with predicted metabolic pathways for fermentation are indicated by black circles. Taxonomic classification at the phylum level of the most abundant classes (*n* > 10) is indicated by the color in the outer ring and is based on the Genome Taxonomy DataBase r214 and the NCBI taxonomy for bacteria and fungi, respectively. Black circles on the phylogeny show support values (SH-aLRT test ≥80% and ultrafast bootstrap ≥95%, each threshold corresponding to an estimated confidence level of 95% [18]). The model used to generate the phylogeny is shown with a scale bar for estimated amino acid substitutions per site. The outgroup (*n* = 20 FAD-dependent oxidoreductase sequences) is collapsed.

As we were primarily interested in NirB-dependent ammonifiers that may contribute to N retention in soil, we identified fermentative nitrite ammonifiers in the phylogeny by assessing the potential for fermentation in bacterial genomes using Microbe Decoder [10] (*n* = 448 genomes in 29 phyla; Fig. 1). Their presence in terrestrial biomes was examined using GraftM [11] and a custom reference package built using the full NirB phylogeny generated in this study. Prior to the downstream analyses, the *nirB* counts were normalized by calculating the ratio between *nirB* counts and the total number of base pairs sequenced in each metagenome, thus reflecting the community-level genetic potential for fermentative nitrite ammonification. Our results show that fermentative nitrite ammonifiers are present in all biomes (Fig. 2a), though in different proportions relative to NrfA-dependent ammonifiers (Fig. 2b) and often with large within-biome variation. They were particularly abundant in Mediterranean forests and desert soils (Fig. 2a), where they dominated the nitrite ammonifying communities (Fig. 2b), and exhibited the lowest prevalence in tundra and in boreal forests and taiga (Fig. 2a). Placement on the NirB phylogeny showed that fermentative nitrite ammonifiers in soils are dominated by a few clades of Actinomycetota (50% of *nirB* counts) and Pseudomonadota (23%; Fig. S1). This limited diversity resulted in minimal differences among biomes (Fig. S2), with Actinomycetota accounting for ~65% of *nirB* counts in Mediterranean forests and desert soils. We note that our approach did not distinguish between facultative and obligate fermenters and that the genomic potential for fermentation does not guarantee that NirB-dependent nitrite ammonification and fermentation are always coupled. Rather, our results reflect differences in genetic potential for fermentative nitrite ammonification across terrestrial biomes.

**Figure 2:**
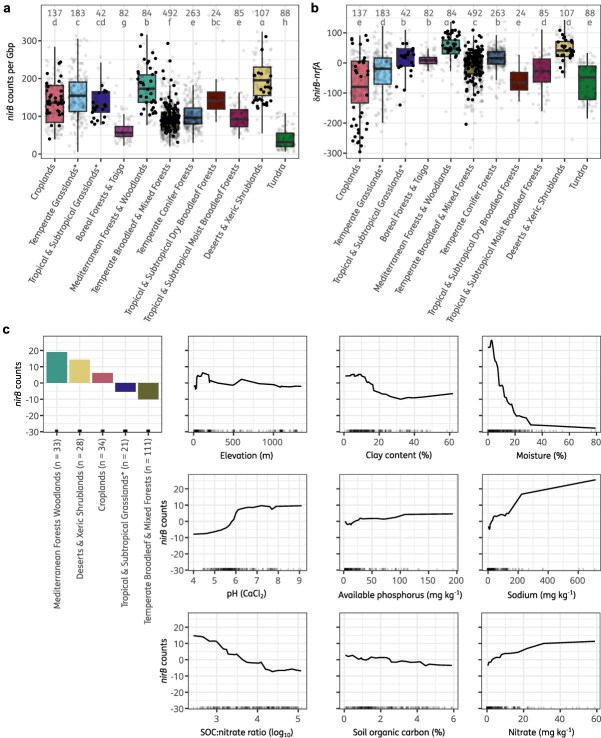
**Abundance and environmental drivers of fermentative nitrite ammonifiers across terrestrial biomes.** (a) Normalized *nirB* counts per biome, calculated as the ratio between *nirB* counts and the total number of base pairs (Gbp) sequenced in each metagenome (*n* = 1587 metagenomes; Kruskal–Wallis test, H(10) = 669, *P* = 3.09 × 10^−137^). (b) Relative importance of the genetic potential for fermentative nitrite ammonification and NrfA-dependent ammonification across terrestrial biomes. The difference in counts between *nirB* and *nrfA* genes (δ*nirB*-*nrfA*) was calculated per metagenome. *nrfA* counts were obtained from a previous study [17]. *nirB* counts were divided by two to account for gene length differences (*nirB*, ca. 3000 bp; *nrfA*, ca. 1500 bp) and values were normalized by the number of base pairs sequenced (*n* = 1587 metagenomes; Kruskal–Wallis test, H(10) = 440, *P* = 2.49 × 10^−88^). Significant differences are denoted with different letters, together with the number of metagenomes representing each biome above the boxplots. Boxes are bounded on the first and third quartiles; horizontal lines represent medians. Whiskers denote 1.5× the interquartile range. Data points corresponding to the metagenomes used in the random forest models are shown as filled circles. (c) Environmental predictors of fermentative nitrite ammonifiers in soil based on random forest models. The analysis was performed on a subset of the metagenomes (*n* = 227) and the number of metagenomes corresponding to each biome is indicated after the biome name. Predictor variables were used to generate accumulated local effects plots, which show the differences in prediction of normalized *nirB* counts (y-axis) compared to the mean prediction along the range of each predictor (x-axis), while accounting for potential correlations amongst predictor values [19]. The effect is centred so that the mean effect is zero. The random forest model was built with 500 trees, 3 features considered at each split and a tree depth set to 8 (variance explained: 50.6%). SOC: Soil organic carbon. ^*^The biome name also includes savannas and shrublands.

Finally, we sought to determine the environmental drivers of fermentative nitrite ammonifiers using random forest modelling on a subset of the metagenomes for which soil metadata relevant to ammonification was available (*n* = 227 metagenomes from the “Biomes of Australian Soil Environments” project [12]; see Table S1 for summary statistics at biome level). Accumulated local effect plots revealed a negative relationship between normalized *nirB* counts and the soil organic carbon to nitrate ratio, which was driven by nitrate rather than carbon content (Fig. 2c). This could reflect conditions in which microorganisms benefit from an intracellular electron sink to reoxidize NADH during fermentation while simultaneously preventing toxic nitrite accumulation, consistent with *nirB* being induced by high nitrate/nitrite levels [13] and the role of NirB in detoxification [14]. Our findings further indicate the existence of ecological differences between fermentative nitrite ammonifiers and NrfA-dependent ammonifiers, as the latter are typically promoted under high carbon-to-nitrate ratio, where high electron donor availability allows nitrite reduction to be coupled to energy conservation via NrfA [3]. Niche differentiation between the two ammonifier types is also supported by the low prevalence of *nrfA* in the genome of fermentative nitrite ammonifiers (8%; Fig. 1) as well as distinct biome preferences (Fig. 2b). The counterintuitive low abundance of fermentative nitrite ammonifiers relative to NrfA-dependent ammonifiers in N-rich soils such as croplands might be explained by the limited role of nitrate content in determining the distribution of these organisms. Indeed, the best predictors of *nirB* counts were moisture and sodium content, with the highest *nirB* predictions associated with dry (threshold at 3.5% moisture) and saline soils (Fig. 2c). This suggests high tolerance to osmotic stress in fermentative nitrite ammonifiers, possibly because they can maintain intracellular redox balance without relying on external electron sinks that may be diffusion-limited in dry or saline soil microenvironments [15]. These results further align with the predominance of Actinomycetota in the *nirB* counts of Mediterranean forests and desert soils, as members of this group are known to have exceptional tolerances to both desiccation and salinity stress [16]. Finally, biome identity remained an important driver of normalized *nirB* counts, even after accounting for other environmental variables, with the random forest predictions aligning well with the patterns observed for the full dataset (Fig. 2a).

In conclusion, this study shows that fermentative nitrite ammonifiers are widespread in soils, and ecologically distinct from NrfA-dependent ammonifiers. Further work aiming to elucidate the physiological roles of NirB in different soil taxa, particularly Actinomycetota and Pseudomonadota, as well as community-level activity rates are needed to determine the conditions under which fermentative nitrite ammonifiers contribute to ammonium pools in terrestrial environments.

## Supplementary Material

Supplementary_material_ycag144

## Data Availability

The data supporting this article, including and the GraftM package for NirB, is available as supplementary material. The methods for constructing the reference phylogeny and conducting the GraftM search are described in detail in [17].
