# Supplementary material for: Fermentative nitrite ammonifiers are abundant in soils and ecologically distinct from NrfA-dependent ammonifiers
Source: ISME Commun. 2026 May 23;6(1):ycag144. doi: 10.1093/ismeco/ycag144 (PMC13308654; doi:10.1093/ismeco/ycag144)
Supplement: Supplementary_material_ycag144 [file supplementary_material_ycag144.pdf]

Supplementary information

Fermentative nitrite ammonifiers are abundant in soils and ecologically distinct from NrfA-dependent ammonifiers

Yuyan Teng and Aurélien Saghaï

Content:

Supplementary Table 1

Supplementary Figures 1-2

Table S1. Summary statistics at the biome level of the variables included in the random forest models (mean ± s.d.). SOC: soil organic carbon.

	Elevation (m)	Clay (%)	Moisture (%)	pH ($CaCl_2$)	Sodium (mg kg^{-1})	Phosphorus (mg kg^{-1})	SOC:nitrate ratio (log_{10})	Nitrate (mg kg^{-1})	Soil organic carbon (%)
Croplands	326 ± 335.1	26 ± 14.6	14.8 ± 12.5	6.99 ± 0.99	104.7 ± 118.1	80.6 ± 44	3.11 ± 0.45	17.6 ± 15	1.79 ± 1.08
Deserts and Xeric Shrublands	477.6 ± 168.2	11.2 ± 7.5	2.8 ± 4.3	6.42 ± 0.77	25.3 ± 32.3	7.1 ± 6.9	3.53 ± 0.44	4.7 ± 7.6	1.22 ± 1.1
Mediterranean Forests Woodlands and Scrub	150.4 ± 91.3	9.6 ± 7.2	5.3 ± 3.9	6.09 ± 0.88	81.5 ± 126.5	5.5 ± 8.1	4.06 ± 0.52	3.3 ± 7.4	1.4 ± 0.82
Temperate Broadleaf and Mixed Forests	579.5 ± 550.8	16.5 ± 11.9	19.1 ± 12.5	5.5 ± 0.66	36.2 ± 76.9	19 ± 16.8	4.09 ± 0.7	4.6 ± 7.4	2.83 ± 1.59
Tropical and Subtropical Grasslands Savannas and Shrublands	125.1 ± 230.2	12.2 ± 8.3	5.4 ± 4.7	5.78 ± 0.46	6.5 ± 4.8	5.8 ± 4.9	4.22 ± 0.32	1.2 ± 1.4	1.5 ± 0.79

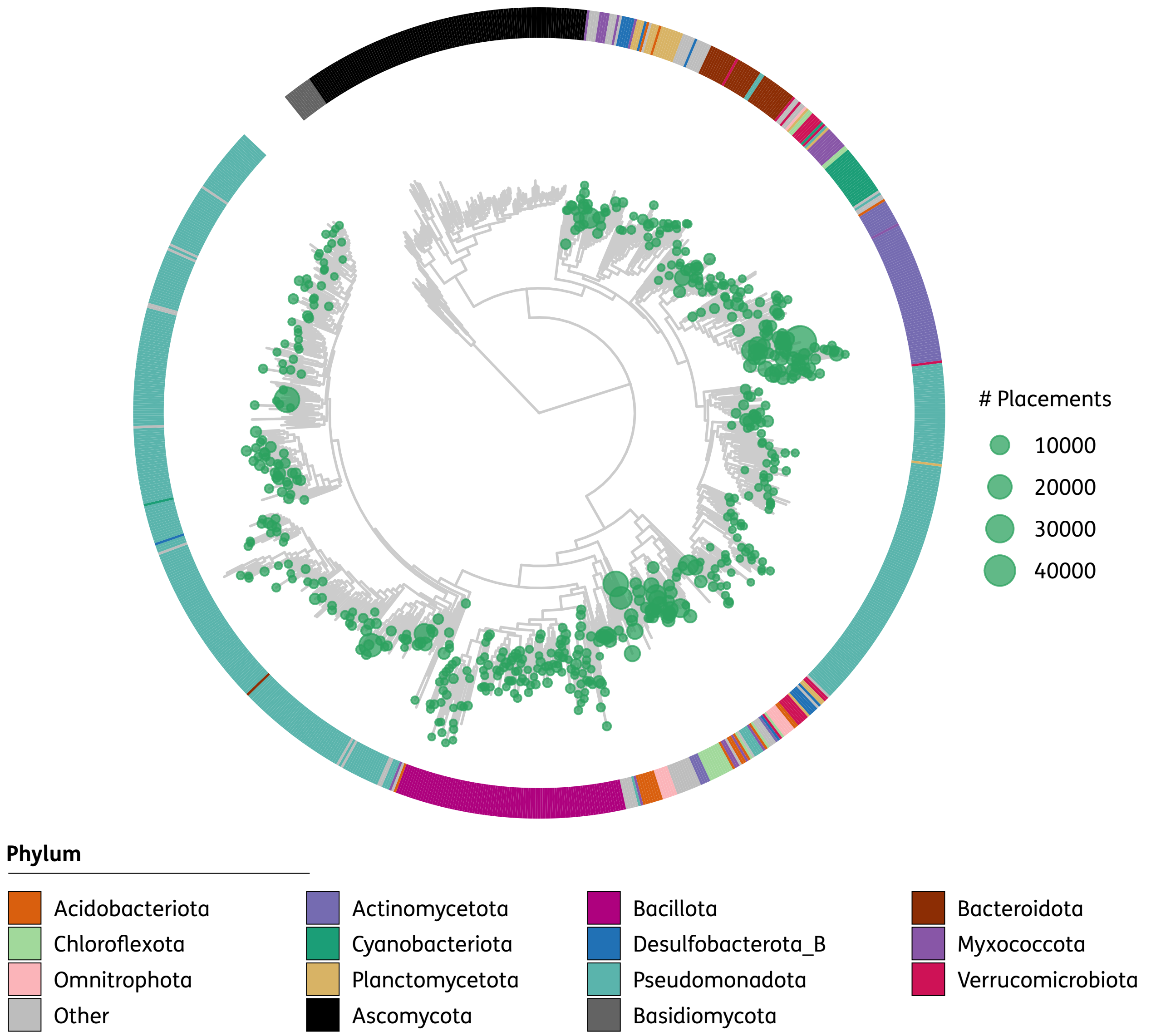

Figure S1. Phylogenetic placements of the metagenomic *nirB* sequence fragments across biomes (for fermentative nitrite ammonifiers only). The size of the dots is proportional to the number of placements. Taxonomic classification at the phylum level of the most abundant classes (n > 10) is indicated by the color in the ring and is based on the Genome Taxonomy DataBase r214 and the NCBI taxonomy for bacteria and fungi, respectively.

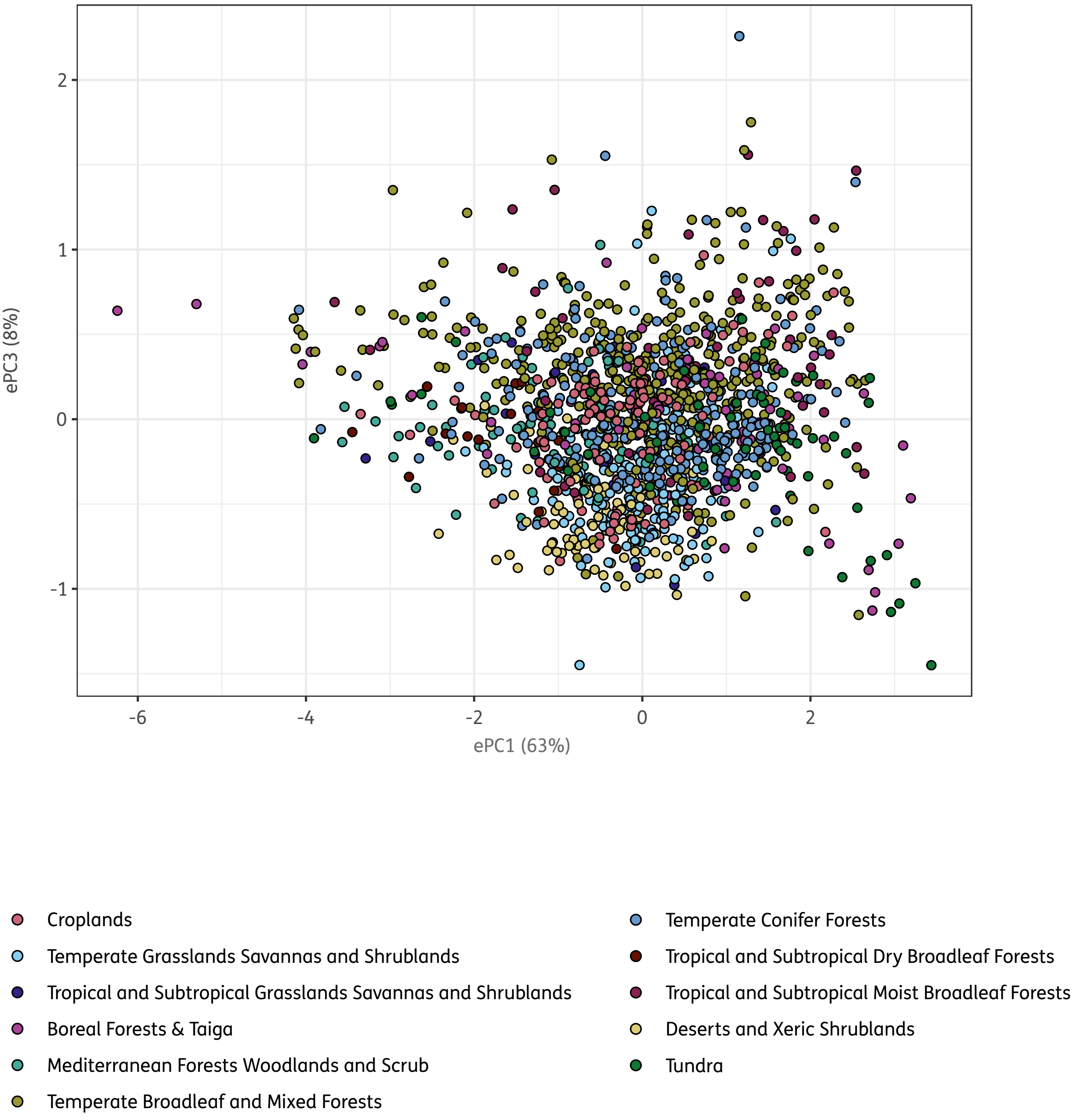

Figure S2. Phylogeny-based community composition across biomes. Edge principal component analysis showing differences in *nirB* community composition between metagenomes grouped into biomes. The ordination was performed on metagenomes with ≥ 20 *nirB* placements (n = 1,420) using GAPPA (https://github.com/lczech/gappa).
